# Severe Tick-Borne Encephalitis (TBE) in a Patient with X-Linked Agammaglobulinemia; Treatment with TBE Virus IgG Positive Plasma, Clinical Outcome and T Cell Responses

**DOI:** 10.1007/s10875-024-01718-5

**Published:** 2024-04-27

**Authors:** Wilhelm Hedin, Peter Bergman, Mily Akhirunessa, Sandra Söderholm, Marcus Buggert, Tobias Granberg, Sara Gredmark-Russ, C. I. Edvard Smith, Aleksandra Pettke, Emilie Wahren Borgström

**Affiliations:** 1 Department of Clinical Microbiology, Karolinska University Hospital, Stockholm, Sweden; 2 Department of Clinical Immunology and Transfusion Medicine, Karolinska University Hospital, Stockholm, Sweden; 3 Department of Laboratory Medicine, Clinical Immunology, Karolinska Institutet, Stockholm, Sweden; 4 Department of Medicine Huddinge, Center for Infectious Medicine, Karolinska Institutet, Stockholm, Sweden; 5 Department of Microbiology, Public Health Agency of Sweden, Solna, Sweden; 6 Department of Neuroradiology, Karolinska University Hospital, Stockholm, Sweden; 7 Department of Clinical Neuroscience, Karolinska Institutet, Stockholm, Sweden; 8 Department of Infectious Diseases, Karolinska University Hospital, Stockholm, Sweden; 9 Department of Laboratory Medicine, Biomolecular and Cellular Medicine, Karolinska Institutet, Stockholm, Sweden

**Keywords:** X-linked agammaglobulinemia, immunodeficiency, tick-borne encephalitis, neutralising antibodies, CD8^+^ T cells, tetramers

## Abstract

**Purpose:**

A patient with X-linked agammaglobulinemia (XLA) and severe tick-borne encephalitis (TBE) was treated with TBE virus (TBEV) IgG positive plasma. The patient’s clinical response, humoral and cellular immune responses were characterized pre- and post-infection.

**Methods:**

ELISA and neutralisation assays were performed on sera and TBEV PCR assay on sera and cerebrospinal fluid. T cell assays were conducted on peripheral blood the patient and five healthy vaccinated controls.

**Results:**

The patient was admitted to the hospital with headache and fever. He was not vaccinated against TBE but receiving subcutaneous IgG-replacement therapy (IGRT). TBEV IgG antibodies were low-level positive (due to scIGRT), but the TBEV IgM and TBEV neutralisation tests were negative. During hospitalisation his clinical condition deteriorated (Glasgow coma scale 3/15) and he was treated in the ICU with corticosteroids and external ventricular drainage. He was then treated with plasma containing TBEV IgG without apparent side effects. His symptoms improved within a few days and the TBEV neutralisation test converted to positive. Robust CD8^+^ T cell responses were observed at three and 18-months post-infection, in the absence of B cells. This was confirmed by tetramers specific for TBEV.

**Conclusion:**

TBEV IgG-positive plasma given to an XLA patient with TBE without evident adverse reactions may have contributed to a positive clinical outcome. Similar approaches could offer a promising foundation for researching therapeutic options for patients with humoral immunodeficiencies. Importantly, a robust CD8^+^ T cell response was observed after infection despite the lack of B cells and indicates that these patients can clear acute viral infections and could benefit from future vaccination programs.

**Supplementary Information:**

The online version contains supplementary material available at 10.1007/s10875-024-01718-5.

## Introduction

X-linked agammaglobulinemia (XLA) is an inborn error of immunity caused by mutations in the gene encoding Bruton’s tyrosine kinase (BTK) [1]. The gene is located on the long arm of the X-chromosome (Xq22.1).There are more than 1000 different mutations in the *BTK* gene that can cause XLA [2]. Patients have very low levels (< 1%) of mature B lymphocytes and are therefore totally unable to produce immunoglobulins. The disease is also called Bruton´s disease and affects 1 in 200.000 male births [3, 4]. Patients with XLA suffer from recurrent bacterial infections from an early age, and are at an increased risk of encephalitis due to enterovirus infection. Other viral infections described are chronic Norovirus infection with fecal shedding [5], and progressive multifocal encephalopathy by JC virus [6].

Studies of the T cell compartment in patients with XLA have shown normal numbers of CD4^+^ and CD8^+^ T cells, but have revealed an impaired function due to a decreased T cell receptor (TCR) repertoire and a reduced number of follicular T helper (Th) cells, Th17 cells, regulatory T cells, and memory T cells [7–11]. Recently, however, a potent T cell response to COVID-19 mRNA-vaccination was detected in patients with XLA [12].

Tick-borne encephalitis (TBE) is a neurological infectious disease caused by the flavivirus tick-borne encephalitis virus (TBEV). The virus is transmitted by infected ticks of the Ixodes spp. Endemic areas are north-eastern Asia [13] and Europe including Russia, the Baltics, Scandinavia as well as Eastern and Central Europe [14]. TBE is an emerging infection, with increasing numbers of cases and geographical expansion in Europe [15, 16], in the past 20 years. In Sweden, the geographic expansion of TBE varies, with a clear endemic focus in Southern Sweden [17]. The Stockholm area is highly endemic, with incidence reports varying between 3 –12/100.000 inhabitants [18, 19]. In 2015, vaccination coverage in Stockholm was reported to be around 53% of the population (fully vaccinated) [18]. Currently, no specific treatment is available.

Typically, TBE is a biphasic disease, with a short flu-like illness followed by meningoencephalitis. The morbidity rate is around 20–30% [20] and mortality rate is 1–2% [21]. Up to 20% of patients suffer from long-term sequelae after recovery, including cognitive and movement impairments [22, 23]. Hypogammaglobulinemia has been associated with lower antibody responses after TBE immunisation [24], but little is known about the clinical picture of TBE infection in these patients.

In this study, we present the clinical picture of a patient with XLA who suffered from severe TBE and received extensive treatment in the intensive care unit (ICU), including plasma transfusions containing TBEV-IgG antibodies and corticosteroids. We describe the clinical outcome and T cell responses in cells collected before and after infection. Finally, we discuss the clinical implications of our findings in relation to diagnostics of and vaccination against TBE.

## Methods

### Clinical data

Patient data were gathered during admission to the Department of Infectious Diseases at Karolinska University Hospital, Stockholm, Sweden. Oral and written informed consent was obtained from the patient.

### Radiology

Brain Computed tomography (CT) and Magnetic Resonance Imaging (MRI) of the brain and spinal cord were performed according to clinical routine at the Department of Neuroradiology, Karolinska University Hospital.

### Laboratory analyses

Peripheral blood specimens and cerebrospinal fluid (CSF) were sent for blood chemistry and microbiological analysis to the Karolinska University Laboratory, Department of Laboratory Medicine, Karolinska University Hospital and to the Public Health Agency of Sweden, according to clinical routines (Fig. 2).

#### TBEV polymerase chain reaction (PCR)

Serum and CSF samples were examined for TBEV. RNA was extracted using a MagLEAD® 12gC extraction robot from PSS (Precision System Science Co., Ltd.). For the detection of TBEV RNA two different PCR systems were used, one that is TBEV specific (TBEV NS3) and another that detects several viruses from the TBEV complex [25]. Primers and probes for TBEV PCR are described in Supplementary Table 1.

#### TBEV enzyme-linked immunosorbent assay (ELISA)

Plasma from healthy donors was tested for the presence of TBEV IgG antibodies, and five units of plasma containing TBEV-specific antibodies were sent to the ward. The analyses were conducted at the routine diagnostic laboratory at the Department of Laboratory Medicine, Clinical Microbiology, Karolinska University Hospital. In brief, the plasma was analysed for TBEV-IgG using a Tecan Freedom EVOlyzer with a Virotech TBE Kit according to the manufacturer’s instructions.

#### TBEV neutralisation assay

A rapid fluorescent focus inhibition test (RFFIT) was used to detect TBEV neutralizing antibodies in the patient’s sera at three different time points: day 1, 9 and 12 [26]. The RFFIT neutralization test was based on the ability of the antibodies in each sample to prevent the propagation of TBEV in Baby hamster kidney-21 (BHK-21) S13 cells. The neutralizing antibody titers were calculated from the serum dilution that reduced the TBEV to one fifty-per-cent-focus-forming-dose (FFD_50_).

#### T cell assays

Samples were collected as part of a parallel study (COVAXID) on which the patient was enrolled and where responses to COVID-19 vaccination were studied in patients with various immunological disorders, including XLA [27]. Blood samples from five healthy and previously TBE-vaccinated controls were obtained from staff at the Department of Infectious Diseases. Peripheral blood mononuclear cells (PBMC) were isolated and stored in freezing medium in an ultralow temperature freezer (liquid nitrogen). The experimental results of all T cell experiments were specifically generated for this study and are presented in Fig. 3.

Briefly, PBMCs were stimulated with overlapping peptides from the TBEV genome (specifically proteins NS5, E and C) to assess TBEV-specific CD4^+^ and CD8^+^ T cells using an activation-induced markers (AIMs) assay in the patient and in healthy controls. For extensive method description see description in Supplementary Material.

## Results

### Case presentation

The patient was a 47-year-old man who regularly attended the Immunodeficiency Unit at the Department of Infectious Diseases at Karolinska University Hospital due to XLA. His disease-causing genetic variant has been previously described [28, 29] and reported to the BTK base registry [30].

He had a long history of recurrent bacterial airway infections, a previous episode of Henoch-Schönlein’s purpura in childhood, as well as meningoencephalitis with transient ataxia in childhood with no sequelae. He was treated with intravenous immunoglobulin (Ig) replacement therapy from seven months of age, which was subsequently changed to subcutaneous Ig replacement therapy (ScIGRT), 80 ml/week. He was never vaccinated against TBE. Fourteen days before seeking medical care the patient had a flu-like illness with fever and malaise, which resolved after four days. A week later the fever reappeared together with a severe headache. The patient had not been aware of any tick-bites.

The initial clinical workup at the Emergency Department at Karolinska University Hospital showed that the patient was alert and fully oriented, Glasgow Coma Scale 15/15 (GCS, see Supplementary Table 3). His body temperature was elevated at 39.0 °C, cardiac auscultation was normal, 89 beats per minute (BPM), blood pressure (BP) was 125/88 mmHg, respiratory auscultation was normal and pO_2_ was 97% without oxygen supplement. Neurological examination showed normal cranial nerve function, but an ataxic gait and the finger-to-nose test showed dysmetria on the left side. The Romberg’s test was negative. Muscle power assessment was normal, as was sensory assessment.

**Fig. 1:**
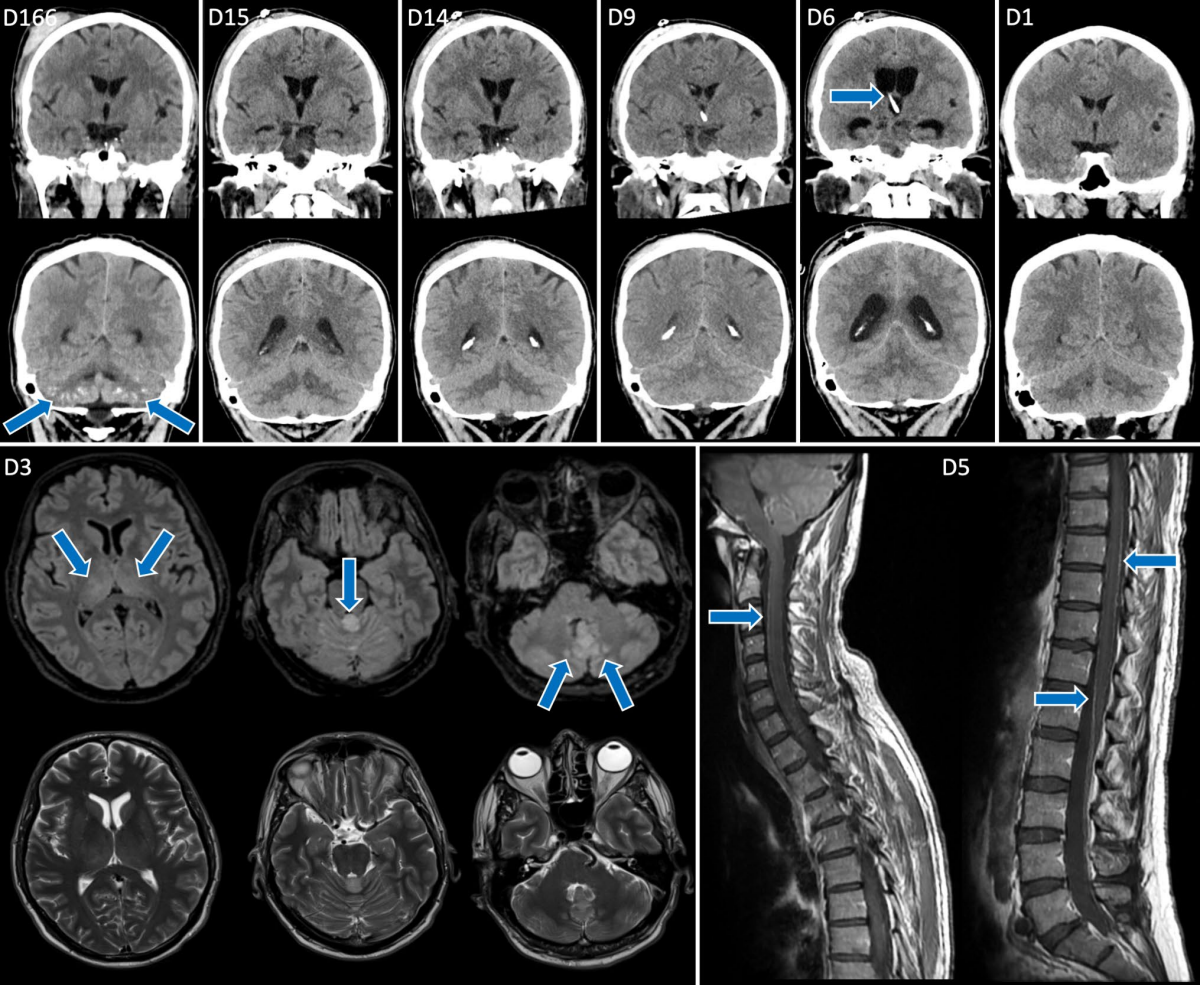
Neuroimaging demonstrating TBE meningoencephalitis. At admission day 1 (D1), brain computed tomography (CT) was normal. Brain magnetic resonance imaging (MRI) on day 3 (lower left panel) revealed edema (arrows) in the thalami bilaterally, superior vermis and medially in the cerebellum. Spinal MRI on day 5 (lower right panel) revealed leptomeningeal enhancement (arrows). On day 6, obstructive hydrocephalus occurred, and the patient was treated with ventricular drainage (arrow), which reconciled the increased pressure (D9), and the drainage could subsequently be removed without hydrocephalus (D14, D15). At a brain CT scan 5.5 months later due to trauma (acute right frontoparietal extracranial hematoma present), extensive brain calcifications in the cerebellar parenchyma had developed (arrows)

Routine blood chemistry and a brain computed tomography (CT) were unremarkable (Table 1, Fig. 1). A lumbar puncture was performed, and the CSF showed pleocytosis and an elevated level of albumin. Due to a clinical suspicion of Herpes Simplex virus (HSV) type 1 encephalitis the patient was started on intravenous acyclovir, 800 mg/dose, three times daily. Routine blood cultures, CSF cultures and PCR tests for Varicella zoster virus (VZV), Enterovirus, HSV 1 and 2 and a blood sample for a TBEV serology were performed.

**Table 1:** Baseline laboratory data

Blood analyses		Reference values		CSF analyses		Reference values	
	result	value	unit		result	value	unit
CRP	2	< 3	mg/L	WBC	107*	0–5	x10E6/L
WBC	6.2	3.5–8.8	x10E9/L	Neutrophils	4*	< 1	x10E6/L
Neutrophils	4.7	1.6–5,9	x10E9/L	Monocytes	103*	0–5	x10E6/L
Monocytes	0.7	0.2–0.8	x10E9/L	Erythrocytes	2*	< 1	x10E6/L
Thrombocytes	140*	145–348	x10E9/L	Lactate	2.1	1.1–2.4	mmol/L
Lymphocytes	0.7*	1.3–3.5	x10E9/L	Glucose	3.1*	> 60%	P-Glucose
Hemoglobin	134	134–170	g/L	Albumin	639*	< 280 mg/L	mg/L
Sodium	134*	137–145	mmol/L	Opening pressure	14.5	< 20	cm H_2_O
Potassium	3.9	3.5–4.6	mmol/L				
Creatinine	81	< 100	µmol/L				
Lactate	1.2	0.5–2.2	mmol/L				
AST	0.41	< 0.76	µcat/L				
ALT	0.57	< 1.1	µcat/L				
Bilirubin	20	> 26	µcat/L				
Pancreas amylase	0.78	0.15–1.10	µcat/L				
Glucose	5.5	4.2–6.0	mmol/L				
S-IgG	13.3	6.7–14.5	g/L				
S-IgA	< 0.03	0.88–4.5	g/L				
S-IgM	< 0.12	0.27–2.1	g/L				

CSF = Cerebrospinal fluid, CRP = C reactive protein, WBC = white blood cells, AST = aspartate aminotransferase, ALT = alanine transaminase, abnormal values marked with *, all baseline values from day 1; S-IgG, S-IgA, S-IgM measured on day 4

On the second day of admission, the patient experienced nausea and vomiting. A neurological examination using the finger-to-nose test now revealed bilateral dysmetria. The PCR tests for HSV1 and 2, VZV and Enterovirus were negative, and acyclovir was discontinued.

**Fig. 2:**
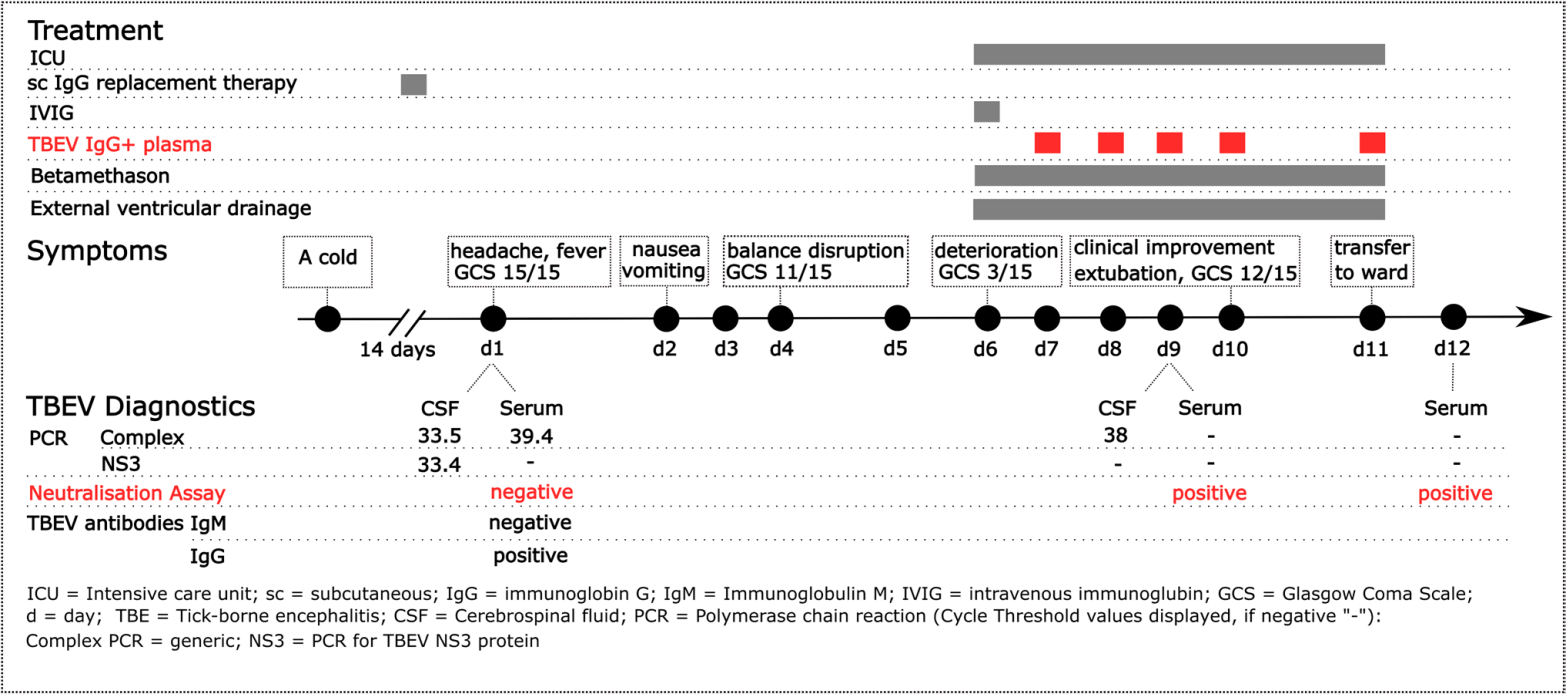
Timeline for symptoms, treatments, and diagnostic results. ICU = intensive care unit, sc = subcutaneous, IgG = immunoglobulin G, IVIG = intravenous immunoglobulin, GCS = Glasgow Coma Scale, d = day, TBE = Tick-borne encephalitis, CSF = cerebrospinal fluid, PCR = polymerase chain reaction, Complex PCR = generic PCR for TBEV proteins; NS3 = specific PCR for TBEV NS3 protein and CT levels = cycle threshold levels

On the third day of admission, brain magnetic resonance imaging (MRI) was conducted and showed edema in both cerebrum and cerebellum (Fig. 1). TBEV-serology showed slightly elevated IgG and negative IgM antibodies (Fig. 2). The serological test result for IgG was not possible to interpret since the patient was receiving scIGRT. Altogether, laboratory findings and imaging were interpreted as consistent with encephalitis and raising the suspicion of TBE.

Four days after admission the patient’s condition deteriorated. He experienced fatigue, disorientation, balance problems and latency of speech; GCS showed 11/15 (E3 + V3 + M5) but was respiratory and circulatory stable. The diagnostic workup was expanded with TBEV-PCR in the serum and CSF.

On the fifth day, spinal MRI showed progression of the infratentorial findings, but without signs of myelitis. The TBEV PCR assay showed a positive result in both the serum and the CSF samples, confirming the diagnosis of TBEV encephalitis.

The next day, the patient’s condition deteriorated further, and he became unconscious. The GCS was 3/15 (E1 + M1 + V1). The patient was intubated and transferred to the neuro-ICU. A follow-up brain CT scan showed increased cerebellar edema, early signs of transforaminal herniation and obstructive hydrocephalus. The patient’s vital signs were affected by bradycardia (40 BPM) and a systolic BP of 170 mmHg. The patient received emergency ventricular drainage and was initially treated with betamethasone 8 mg twice daily and received additional intravenous immunoglobulin (IVIG) replacement therapy (100 mg/ml) 300 ml with the intention to help clear the infection.

On the seventh day of admission the patient´s status was still critical and a decision was made to give the patient rescue therapy in the form of plasma containing specific TBEV antibodies (IgG) on vital indication. Plasma units from healthy Swedish donors were sent to the Department of Clinical Microbiology and tested for specific TBEV antibodies. One unit of TBEV IgG-positive plasma was administered daily for the following five days.

A test for neutralising antibodies against TBEV using pre- and post-treatment sera showed seroconversion for neutralising antibodies after treatment. The patient started to show signs of neurological improvement and was extubated after four days in the ICU. He displayed no adverse effects to the treatment with plasma, such as inflammation or allergic reaction. Due to suspicion of ventilator-associated pneumonia, intravenous cephalosporin treatment was started after the patient had been extubated. One day later, he was transferred from the ICU to a ward at the Department of Infectious Diseases.

Since the XLA patient was part of another study [12], we had access to PBMC samples collected before and three and 18 months after the infection. Notably, robust CD8^+^ T cell responses were observed for all three peptide pools at three- and 18-months post-infection. As expected, the CD4^+^ T cells generated strong positive responses to the peptide pool derived from the E protein in the healthy vaccinated controls. In contrast, CD8 + T cells showed limited responses to the same peptide pool following vaccination.

**Fig. 3:**
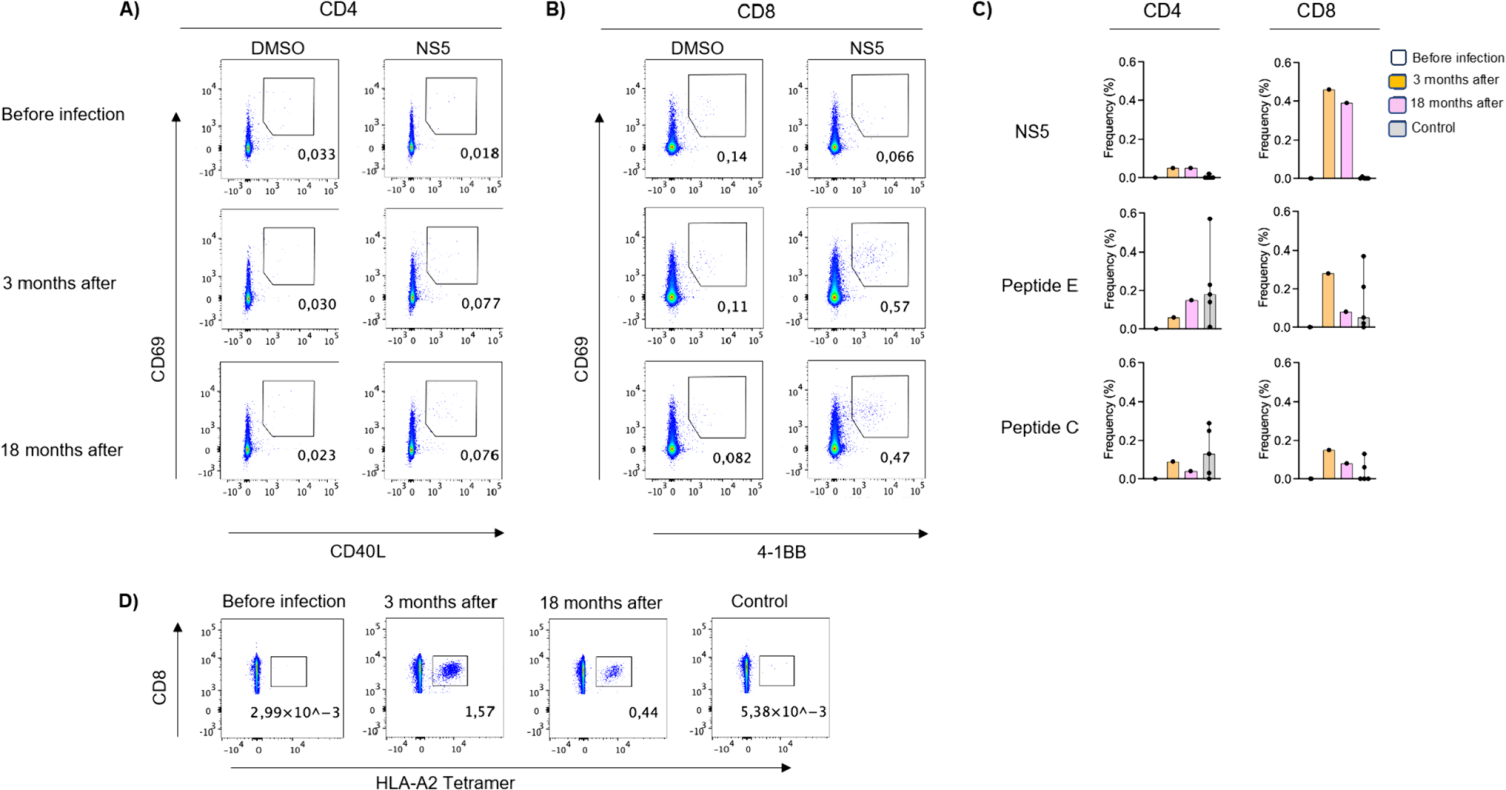
CD4^+^ and CD8^+^ T cell responses to tick-borne encephalitis virus in a patient with XLA. Cluster of differentiation = CD, Dimethyl sulfoxide = DMSO, Non-structural protein 5 = NS5, Ligand = L, Human leucocyte antigen = HLA. Peripheral blood mononuclear cells (PBMCs) were incubated in the presence of three different Tick-borne encephalitis virus (TBEV) peptide pools (NS5, peptide E and peptide C) for 12 h at 37 °C. **A)** Antigen-specific memory CD4^+^ T cell response was measured by the proportion of CD69^+^CD40L^+^ co-expressing cells and **B)** co-expression of CD69^+^4-1BB^+^ was considered as antigen-specific memory CD8^+^ T cells. **C)** Bar plots showing the frequency (percent) of memory CD4^+^ T cells and memory CD8^+^ T cells in response to three TBEV peptide pools from the X-linked agammaglobulinemia (XLA) patient before, three months after and 18 months after TBEV infection. Memory T cell frequency was only measured for the five healthy vaccinated controls at one time point. The frequency of the T cells obtained in di-methyl sulfoxide (DMSO) treated PBMC cultures were used as a control to measure both CD4^+^ and CD8^+^ T cell response to the peptide pools. **D)** Representative flow cytometry plots showing HLA-A2 tetramer-specific memory CD8^+^ T cells in the XLA patient before, three and 18 months after TBEV infection as well as in one vaccinated healthy control

To further support an antigen-specific CD8^+^ T cell response to TBEV infection, we stained PBMCs from the patient with TBEV-restricted HLA class 1 tetramers before and after the infection. These results confirmed the strong responses in the patient after infection, supporting the conclusion that TBEV infection could induce a potent T cell response also in the absence of mature B cells (Fig. 3).

The patient was discharged five weeks later to a neurological rehabilitation clinic where he stayed for around 1.5 months. At clinical follow ups 4, 12 and 24 months later the patient had made a good physical recovery and was going on long walks, but still had cognitive problems and had not been able to return to work. A brain CT scan performed due to head trauma after a fall 5.5 months after admission, notably revealed that the patient had developed extensive calcifications in the cerebellum as a post-infectious sequela.

## Discussion

In addition to respiratory infections, patients with XLA can contract severe viral infections, where enteroviral infection with meningoencephalitis is a dreaded complication [31].

The patient presented in this study had a primary immunodeficiency and contracted a severe form of viral meningoencephalitis – TBE. He was TBEV-PCR positive in both serum and CSF in the second phase of his disease. Viral persistence in serum and CSF is unusual and might indicate a lack of virus neutralisation during the first peripheral disease phase [32]. Chemotherapy in relation to hematological cancer treatment has previously been associated with prolonged viremia [33]. Severe TBE has also been associated with secondary immunosuppression as illustrated by a case of fatal TBE in a boy treated with etoposide and corticosteroids during the viraemic phase [34].

Although there is no established correlate of protection for TBEV, neutralising antibodies are considered the most important mechanism of protection [35]. Titers of ≥ 10 IU/ml have been considered as a reliable protection level in clinical studies investigating the efficacy of vaccination [36]. Also, it has previously been shown that TBEV-specific antibody content of IVIG had a highly neutralising effect in cell culture and correlated with protection from disease in mice [37]. Interestingly, even though the patient received subcutaneous IgG replacement therapy (Hizentra, CSL Behring), no neutralising antibodies against TBEV were detected in the patient’s serum at disease onset.

Importantly, the seroprevalence of TBEV antibodies in blood donors and immunoglobulin replacement products can differ widely, mainly due to the endemic nature of the disease and varying vaccination coverage in donor populations [38, 39]. Additionally, due to the manufacturing process of IVIG with variable sources of human plasma, a lot-to-lot variation in the levels of antibodies against various pathogens are expected and have been reported [40]. Considering the role of TBEV as an emerging pathogen, including TBEV in the panel of infectious agents for which antibody content needs to be declared by manufacturers, may benefit immunodeficient patients, such as those with common variable immunodeficiency or those undergoing rituximab treatment.

The patient was treated in the ICU and received external ventricular drainage. Notably, the clinical course of the patient started to improve after treatment was initiated with plasma from healthy donors containing antibodies against TBEV. Moreover, following this plasma administration, neutralising antibodies with a titer of 10 IU/ml were detected in the patient’s serum, indicating the presence of a protective level of antibodies. The viral load detected by PCR in CSF was reduced from cycle threshold level 33 to 38, further indicating viral clearance. It is unclear if antibodies administered through plasma treatment will pass through the blood brain barrier (BBB). However, previous studies on mice have found BBB disruption and high TBEV viral load present in the brain [41]. An additional study has proposed increased permeability of the BBB in patients with TBE. [42], suggesting that peripherally administered TBE-specific IgG potentially could pass through the BBB and contribute to intrathecal elimination of the virus.

It is evident that modern intensive care with external ventricular drainage was a directly life saving measure. The latter improvement of the clinical course could be due to a combination of factors such as corticosteroids and plasma containing specific TBEV antibodies (IgG). The corticosteroids did not seem to have an obvious adverse effect on viral clearance in an already immunocompromised patient, and TBEV IgG positive plasma was administered concomitantly as the corticosteroids and may thus have protected against adverse effects. The corticosteroids could similarly have protected this particular patient from allergic reactions or inflammatory complications due to antibody-dependent enhancement, which has previously been described after vaccination against other flaviviruses [43].

We had access to cells collected before and post-infection, which enabled detailed studies to be conducted of CD4^+^ and CD8^+^ T cell responses to TBEV-infection in the patient. Our results revealed a strong CD8^+^-response at three months, which was still present at 18 months, suggesting long-lasting immunity. The patient’s CD4^+^ response was weak. These findings are in line with previous studies where a strong CD8^+^ response has been shown after TBE-infection [44], whereas TBE vaccination induces CD4^+^ T cell responses, but no memory CD8^+^ T cell responses [45]. Our data suggest that the T cell pool in the patient with XLA produces long-lasting TBEV-specific CD8^+^ T cells, similar to other infected individuals. However, it is still unclear whether this response is sufficient to provide clinical protection upon re-exposure to the virus.

In conclusion, this case has several clinical implications. While demonstrating that patients with hypogammaglobulinemia can suffer from a severe TBEV-infection, it shows that treatment with plasma with detectable levels of TBEV-specific IgG can provide a patient with neutralising titers and potentially facilitate viral clearance. Most importantly, such treatment was not associated with any adverse events in this particular case. This implies that even treatment with monoclonal antibodies might be a future therapeutic option both as prophylaxis and during infection, while considering the addition of corticosteroids to avoid adverse effects. Treatment with TBEV monoclonal antibodies has previously been demonstrated to be effective in mice [46], and monoclonal antibodies have been used successfully for prevention and treatment of other viral infections like respiratory syncytial virus (RSV) and COVID-19 [47, 48]. Thus, this case offers a promising foundation for researching therapeutic options, regarding treatment of immunocompromised patients, with severe TBE-infection and suspected ongoing viral replication.

Finally, the T cells of the patient presented in this study responded strongly to TBEV-infection and lead to immunological memory. The strong CD8^+^T cell response post-TBEV infection raised the question as to whether next-generation mRNA vaccines, proven to boost cellular immunity in immunodeficient patients [49], should also be employed for TBE, potentially benefiting those with XLA and other hypogammaglobulinemic disorders.

Further studies are warranted to elucidate the potential benefits of this therapeutic approach and its immunological implications.

### Supplementary Information

Below is the link to the electronic supplementary material.

Supplementary file1 (DOCX 48 KB)

## Data Availability

No datasets were generated or analysed during the current study.
