# Supplementary material for: Severe Tick-Borne Encephalitis (TBE) in a Patient with X-Linked Agammaglobulinemia; Treatment with TBE Virus IgG Positive Plasma, Clinical Outcome and T Cell Responses
Source: J Clin Immunol. 2024 Apr 27;44(5):116. doi: 10.1007/s10875-024-01718-5 (PMC11055791; doi:10.1007/s10875-024-01718-5)
Supplement: Supplementary file 1 — Supplementary file1 (DOCX 48 KB) [file 10875_2024_1718_MOESM1_ESM.docx]

**Hedin et al. Supplementary material**

**Supplementary table 1:** Primers and probes used in TBE PCR

| **Target** | **Primer/Probe** | **Sequence 5’ to 3’** | **Reference** |
| --- | --- | --- | --- |
| TBEV NS3 | TBE - P | CACAGAGTCCTCCC-FAM-MGB |  |
|  | TBE - F | TGGACATGCACCCAGGCT |  |
|  | TBE - R | GCTCCATTTCYTTGAGYACCAC |  |
| TBE Complex | TBE - P | CCACCATCACCCAGAC-FAM-MGB | Modified from (1) |
|  | TBE - F | GCGGTTCTTGTTCTCCCTGA |  |
|  | TBE - R | CACACATCACCTCCTTGTCAGAC |  |

**Method description: T-cell-assays:**

Peripheral blood mononuclear cells (PBMC) were isolated and stored in freezing medium in an ultralow temperature freezer (liquid nitrogen). For flow cytometry staining, frozen PBMCs were thawed and washed with Roswell Park Memorial Institute (RPMI) complete medium with DNase (10U/mL; Sigma-Aldrich) to completely remove dimethyl sulfoxide (DMSO). For tetramer staining, 2x10^6^ cells were washed with 1x phosphate-buffered saline (PBS) and incubated with Dasatinib (Stemcell, catalogue number 73082) in PBS for 10 minutes at room temperature. Thereafter, the cells were mixed with an equal amount of two human leukocyte antigen (HLA) class 1 tetramers (A*02:01-PE (sequence: ILLDNITTL, (2)) and B*07:02-BV421 (sequence: LPLGHRLWL, (3)) by pipetting and were incubated for a further 20 minutes at room temperature. The cells were then washed twice with 1x PBS and stained using LIVE/DEAD™ Fixable Aqua Dead Cell Stain Kit (Life Technologies, catalogue number L34957) for 10 minutes at room temperature. After live-dead staining, the cells were incubated for 15 minutes at 37 °C with antibodies for surface chemokine receptors. The cells were then incubated with a cocktail of antibodies for cell surface markers for another 30 minutes without being washed in between. Following this, the cells were washed twice with a Fluorescence Activated Cell Sorting (FACS) buffer and then fixed with a fixation buffer (BD, catalogue number 554655) at +4 °C for 15 minutes. The cells were subsequently washed once with PBS and resuspended in 200 µl of PBS and acquired in a flow cytometer (BD Symphony A3, BD Biosciences, USA).

Peptides: Peptide pools consisting of 15-mers overlapping with 11 amino acids from 3 different proteins (non-structural protein 5 (NS5), peptide E (envelope protein), and peptide C (capsid protein) found in TBEV (Uniprot, P14336 POLG_TBEVW) were utilized to stimulate PBMCs from the XLA patient and controls. These peptides were reconstituted in DMSO, pooled, and diluted to stock concentrations of 100 μg/ml in (PBS) and stored at −20 °C. Before adding to the PBMC cultures, peptides pool stocks were diluted in RPMI complete medium to the final concentration of 0.5 μg/ml.

Activation-induced markers (AIMs) assays: AIMs assay was performed according to the method described previously (4). Briefly, one million PBMCs were cultured per well after thawing from liquid nitrogen quickly; resuspending them in RPMI 1640 complete medium in the presence of DNase I and rested for 3h in 96-well U-bottom plates (Corning) at 37 °C. Before adding TBEV peptide pools (0.5 μg/ml per peptide), the cells were incubated for 15 minutes at 37 °C in the presence of anti-CD40 (unconjugated) antibody (Miltenyi Biotec). DMSO (0, 1%) (Sigma-Aldrich) was used as negative control to TBEV peptides. Cells were harvested after 12h, washed in FACS buffer (PBS supplemented with 2% FBS and 2 mM ethylenediaminetetraacetic acid (EDTA)) and stained with anti-CC-chemokine receptor (CCR7)/allophycocyanin (APC)-Cy7 for 15 min at 37 °C. Live/Dead Aqua (Life Technologies) was utilized to identify live cells. Staining with a cocktail of antibodies for other relevant surface markers was done for 30 min at room temperature. Stained cells were washed, fixed, and acquired in the flow cytometer (BD FACS Symphony A3, BD Biosciences). The gating strategy followed here was explained in our earlier publication (4). All the antibodies and reagents used in flow cytometry experiments are provided in Supplementary Table 2.

**Supplementary Table 2: Antibodies used in T cell assays**

| Antibody | Conjugate | Clone | Dilution | Catalog number | Company |
| --- | --- | --- | --- | --- | --- |
| Live-Dead Aqua | - | - | 3:5000 | L34957 | Thermo Fisher |
| CD3 | BUV805 | UCHT1 | 1:50 | 612895 | BD |
| CD4 | BUV496 | SK3 | 1:25 | 612936 | BD |
| CD8 | BUV395 | RPA-T8 | 1:250 | 563795 | BD |
| CD45RA | BV570 | HI100 | 1:200 | 304132 | BioLegend |
| CCR7 | APC-Cy7 | G043H7 | 1:50 | 353212 | BioLegend |
| CD28 | BUV563 | CD28.2 | 1:50 | 741392 | BD |
| PD-1 | BUV615 | EH12.1 | 1:50 | 612991 | BD |
| CD38 | APC-R700 | HIT2 | 1:50 | 564979 | BD |
| CD40L (CD154) | BV421 | 24-31 | 1:25 | 310824 | BioLegend |
| CD14 | BV510 | M5E2 | 1:100 | 301842 | BioLegend |
| CD19 | BV510 | HIB19 | 1:100 | 302242 | BioLegend |
| HLA-DR | BV605 | G46-6 | 1:33 | 562845 | BD |
| CD69 | BV650 | FN50 | 1:50 | 310934 | BioLegend |
| TIGIT | BV786 | 741182 | 1:200 | 747838 | BioLegend |
| CD95 | PE/Dazzle594 | DX2 | 1:50 | 305634 | BioLegend |
| CD127 | PEcy5 | A019D5 | 1:100 | 351324 | BioLegend |
| 4-1 BB (CD137) | PEcy7 | 4B4-1 | 1:25 | 309818 | BioLegend |
| CD40 (unconjugated) | - | HB14 | 1:200 | 130-094-133 | Miltenyi Biotec |

FlowJo software (version 10) was used for data analyses.

CD=cluster of differentiation, CCR= C-C chemokine receptor, PD=programmed cell death protein 1, HLA=human leucocyte antigen, TIGIT=T cell immunoreceptor, BUV=brilliant ultraviolet, BV=brilliant violet, PE=phycoerythrin, BD=Becton Dickinson.

**Supplementary table 3. Glasgow coma scale (5)**

| **Eye opening (E)** | **Verbal response (V)** | **Motor response (M)** |
| --- | --- | --- |
| 1. Spontaneous | 1. Oriented | 1. Obeys commands |
| 2. To speech | 2. Sentences | 2. Localizes pain |
| 3. To pain | 3. Words | 3. Flexion/withdrawal to pain |
| 4. No response | 4. Sounds | 4. Abnormal flexion to pain |
|  | 5. No response | 5. Extension to pain |
|  |  | 6. No response |

Score the best level of response seen for each component.

**References**

1. Schwaiger M, Cassinotti P. Development of a quantitative real-time RT-PCR assay with internal control for the laboratory detection of tick borne encephalitis virus (TBEV) RNA. J Clin Virol. 2003;27(2):136-45.

2. Blom K, Braun M, Pakalniene J, Dailidyte L, Béziat V, Lampen MH, et al. Specificity and dynamics of effector and memory CD8 T cell responses in human tick-borne encephalitis virus infection. PLoS Pathog. 2015;11(1):e1004622.

3. Lampen MH, Uchtenhagen H, Blom K, Varnaitė R, Pakalniene J, Dailidyte L, et al. Breadth and Dynamics of HLA-A2- and HLA-B7-Restricted CD8(+) T Cell Responses against Nonstructural Viral Proteins in Acute Human Tick-Borne Encephalitis Virus Infection. Immunohorizons. 2018;2(6):172-84.

4. Gao Y, Cai C, Wullimann D, Niessl J, Rivera-Ballesteros O, Chen P, et al. Immunodeficiency syndromes differentially impact the functional profile of SARS-CoV-2-specific T cells elicited by mRNA vaccination. Immunity. 2022;55(9):1732-46.e5.

5. Teasdale G, Maas A, Lecky F, Manley G, Stocchetti N, Murray G. The Glasgow Coma Scale at 40 years: standing the test of time. Lancet Neurol. 2014;13(8):844-54.
